# Exploring Inner Strength in People Aging With Serious Illness and Cognitive Impairment

**DOI:** 10.1093/geroni/igaa057.2818

**Published:** 2020-12-16

**Authors:** Brianna Morgan

**Affiliations:** University of Pennsylvania, Philadelphia, Pennsylvania, United States

## Abstract

Inner strength is a psychological resource that supports people as they move through challenging life events. Understanding how people living with serious illness conceptualize inner strength is key in informing person-centered healthcare. This concept analysis used dimensional analysis methods to explore the nature of inner strength in people aging with serious illness to define a situation specific theory. 1212 abstracts and articles from the published literature were reviewed. 30 articles were included and analyzed as data sources. The resulting explanatory matrix conceptualized inner strength in people as an inward and outward process of “meeting me” – an authentic version of oneself – in the context of shifting health. Of note, while other serious illnesses were represented, no literature focused on dementia. Understanding inner strength in people with dementia is the next step for further inquiry. A pilot study exploring feasibility and informing further research is planned.

